# Feasibility of a minimal dataset for adults with acquired brain injury in Dutch healthcare practice

**DOI:** 10.1371/journal.pone.0235085

**Published:** 2020-06-22

**Authors:** Anne-Fleur Domensino, Jolanda C. M. van Haastregt, Ieke Winkens, Coen A. M. van Bennekom, Caroline M. van Heugten

**Affiliations:** 1 School for Mental Health and Neuroscience, Faculty of Health, Medicine and Life Sciences, Maastricht University Medical Center, Maastricht, The Netherlands; 2 Limburg Brain Injury Center, Maastricht, The Netherlands; 3 Care and Public Health Research Institute, Faculty of Health, Medicine and Life Sciences, Maastricht University Medical Center, Maastricht, The Netherlands; 4 Department of Neuropsychology and Psychopharmacology, Faculty of Psychology and Neuroscience, Maastricht University, Maastricht, The Netherlands; 5 Department of Research and Development, Heliomare Rehabilitation Center, Wijk aan Zee, The Netherlands; 6 Coronel Institute of Occupational Health, Amsterdam Public Health Research Institute, Amsterdam University Medical Center, location AMC, The Netherlands; Chinese Academy of Medical Sciences and Peking Union Medical College, CHINA

## Abstract

**Objective:**

Data collection in the field of acquired brain injury (ABI) lacks uniformity due to the broad spectrum of available measurement instruments, leading to incomparability of data and the need for patients to ‘repeat their story’. To pursue uniform data collection, an ABI-specific minimal dataset (MDS-ABI) is currently under development. The current study aimed to assess the feasibility (performance according to protocol, user opinion, potential implementation barriers, and suggested improvements) of the MDS-ABI in clinical settings.

**Methods:**

A mixed-methods approach was used in a range of healthcare sectors for persons with ABI. Clinicians of several relevant disciplines within these sectors were asked to administer the MDS-ABI to five patients. Subsequently, feasibility according to clinicians was assessed by means of a paper questionnaire about every administration and an online questionnaire about the feasibility in general. Feasibility according to patients was assessed with a paper questionnaire and think aloud interviews.

**Results:**

Thirteen clinicians and 50 patients were included. In general, the MDS-ABI performed according to protocol. Both clinicians and patients were overall satisfied with the content of the MDS-ABI. The Cumulative Illness Rating Scale was regarded incomprehensible, leading to missing data. Further, clinicians indicated that the MDS-ABI would not be suitable for all ABI-patients, as some are incapable of self-report due to potential cognitive problems, communicative problems, fatigue, perceptual problems, or impaired awareness of deficits.

**Conclusion:**

The MDS-ABI is a promising tool for obtaining core information on ABI-patients. The MDS-ABI will be adjusted according to the suggestions. For patients who are incapable of self-report, a proxy-reported version of the self-reported part was developed.

## Introduction

As research into the long-term consequences of acquired brain injury (ABI) continues to grow, the body of collected patient data expands accordingly. However, there is a broad array of outcome domains and available measurement instruments [1–3] leading to data that are difficult to aggregate and therefore, compare. This incomparability of research outcomes is a frequently reported limitation of systematic reviews and meta-analyses in the field of ABI [4–6]. Similarly, clinical assessment protocols for diagnosis, prognosis, and outcome differ across healthcare sectors and disciplines. Since ABI is a chronic condition, patients often receive care in various healthcare settings, resulting in them having to keep ‘repeating their story’ [7]. Consequently, clinicians may needlessly collect data that has already been collected, or use different instruments for measuring the same domains.

In an effort to facilitate uniform data collection in the field of ABI and to reduce the administrative burden on patients and clinicians, an ABI-specific minimal dataset (MDS-ABI) is under development. The MDS-ABI is a set of standardised measurement instruments that is used to obtain an overall image of persons with ABI across healthcare sectors and disciplines, which is usable in both the acute and chronic phase of the condition.

The first version of the MDS-ABI was composed using a three-round Delphi procedure with experts on the development/evaluation of measurement instruments used with persons with ABI [8] and consists of one part that is completed by a healthcare professional, and a second part that acts as a Patient Reported Outcome Measure (PROM). A PROM is filled in by the patient, is thus not influenced by the interpretation of the administrator, and is therefore considered a valuable addition to evaluate patients’ functional status [9].

Although the importance of developing data shaping initiatives such as minimal datasets is increasingly recognised by research authorities, evaluating their clinical feasibility is equally important to enhance implementation. Feasibility can be defined as the extent to which a tool or intervention can be applied to the practice it was designed for. As a concept, it is operationalised in a variety of ways. Within the field of acquired brain injury, feasibility assessment is largely performed in the context of evaluation of the intervention process and is therefore often aimed at evaluating the content, intensity, duration, patient adherence, performance according to protocol, and timing of a treatment [10–14]. Such studies often adopt a mixed-method design, allowing for in-depth exploration of patient and clinician experiences in a smaller sample. A frequently used model for process evaluations of interventions is the model by Saunders et al. [15], distinguishing key elements of feasibility such as fidelity to the intervention, dose (delivered and received), and the contextual factors that can influence success of implementation. To the best of our knowledge, no studies have been performed to evaluate the feasibility of minimal datasets and consequently, there is no comparable framework for this specific purpose. We therefore explored operationalisations of the feasibility elements as formulated by Saunders et al. in the context of minimal datasets.

Some guidelines exist on evaluating the feasibility of single questionnaires. Recently, the Consensus-based Standards for the selection of health Measurement Instruments initiative presented guidelines for selecting outcome measurement instruments to be included in Core Outcome Sets [16]. These standards concern the selection of instruments on the basis of the methodological quality of the instrument. This was, however, part of our Delphi procedure in which the instruments for the MDS-ABI were selected [8]. The current study addresses the applicability of the MDS-ABI for which a number of feasibility items of the standards could be selected, such as comprehensibility, interpretability, and required administration time. Moreover, Moores and colleagues developed an instrument to evaluate the feasibility of single questionnaires in healthcare practice [17], covering very similar concepts. Some of the items of the questionnaire are also applicable to evaluate the feasibility of a set of measurement instruments instead of single questionnaires, such as questions about relevance of the set of questionnaires to the condition and overall question comprehensibility.

The current study evaluated the feasibility of the first version of the MDS-ABI with patients and healthcare professionals in a variety of sectors and disciplines in healthcare for persons with ABI in the Netherlands. Feasibility was investigated using those elements of the process evaluation framework proposed by Saunders et al. [15] that were applicable to the current study: 1) performance of the MDS-ABI according to protocol (fidelity, dose delivered), 2) the opinion of both clinicians and persons with ABI on the MDS-ABI (dose received, satisfaction), and 3), potential barriers to implementing the MDS-ABI in healthcare practice and suggested improvements (context). The feasibility elements were operationalised using feasibility questions as formulated by Prinsen et al. [16] and Moores et al. [17], supplemented with questions that are specific to the use of a minimal dataset in clinical practice.

## Methods

### Ethical approval

The current study was assessed by the ethics committee of the Maastricht University Medical Center (MUMC) (reference number 2017–0040) and the following statement applied: "We are pleased to confirm that the Medical Research Involving Human Subjects Act (WMO) does not apply to the above-mentioned study and that an official approval of this study by our committee is not required." Written informed consent was obtained from all participants.

### Design

This multicentre mixed method study was designed according to the framework for process evaluations by Saunders and colleagues [15]. We used a convergent parallel design [18], meaning that both qualitative and quantitative data are collected simultaneously and prioritised equally [19]. Data were collected between June 2018 and January 2019.

### Participants

The authors approached clinicians who were employed at healthcare institutions that offer care to ABI-patients throughout the Netherlands within their professional network. In order to warrant the opportunity to capture inter-personal differences within similar settings, we wanted to include more than one clinician per institution. With respect to restricting the time spent on research for participating clinicians, we decided to include no more than two clinicians per institution. Due to the partly qualitative nature of our study, we expected this sample to reach data saturation. Therefore, we aimed to include a total of 12 clinicians, employed over 12 different institutions; two institutions within each healthcare sector for adults with ABI (hospital, rehabilitation care, care for elderly persons, mental health care, care for people with disabilities, and ambulatory/community-based care). All types of institutions that offer care specific to acquired brain injury were eligible for this study. There were no further requirements for institution enrolment.

#### Clinicians

Inclusion criteria for clinicians were: being a certified healthcare professional employed at one of the selected institutions, using outcome measures in daily practice, and *performing clinical activities (such as diagnostic and therapeutic processes) among* persons with ABI. A purposive sampling technique was employed in order to involve all relevant disciplines (physicians, psychologists/behaviourists, allied healthcare professionals). All clinicians were asked to use the MDS-ABI with five patients each and evaluate its feasibility. In the case clinicians cared for more than five eligible patients, they were instructed to select a sample that was representative of their patient population.

#### Patients

Eligible patients were inpatients or outpatients of one of the selected 12 institutions. Inclusion criteria were: having any type of medically confirmed ABI (e.g. stroke, traumatic brain injury), being able to provide informed consent, being at least 18 years old, being under the care of a participating clinician, having sufficient comprehension of Dutch language based on clinical judgement, and being capable of filling in the questionnaire themselves or with assistance (orally or in writing). There were no restrictions with regard to injury duration. Patients who acquired brain injury due to neurodegenerative or progressive diseases (e.g. Parkinson's disease, Alzheimer's disease, Multiple Sclerosis) were excluded.

### The MDS-ABI

The MDS-ABI is a paper questionnaire consisting of two parts. Part A is administered by a clinician about their patient and consists of validated questionnaires and screening questions on injury characteristics, communicative functioning, comorbidities, and performance on activities of daily living. Furthermore, it contains a cognitive screening test that is administered to the patient by the clinician. Part B is filled in by the patient alone or with help from a relative or clinician. Patients are explicitly instructed to choose their own answers. Part B asks about demographic information, emotional functioning, experienced social support, energy and participation. Both part A and part B feature general instructions at the beginning of the questionnaire and, when applicable, specific instructions for completing the concerned measurement instrument.

#### Part A

Injury characteristics (date of brain injury, type of brain injury, previous brain injury, hospital stay and discharge destination) are retrieved from the patient’s file. To give an indication of communicative functioning, the clinician fills in a screening question (yes/no) regarding communicative difficulties with the opportunity to elaborate/explain their answer. The Cumulative Illness Rating Scale (CIRS) [20] is used to assess comorbidity [21]. Cognitive functioning of the person with ABI is screened by the clinician using the Montreal Cognitive Assessment (MoCA) [22]. The expected administration time for part A is 25–40 minutes [8].

#### Part B

Demographic characteristics (date of birth, gender, living situation and type of household) are filled in by the patient. Emotional functioning (emotional distress) is assessed using the Hospital Anxiety and Depression Scale (HADS) [23]. Energy (fatigue) is assessed using the Fatigue Severity Scale (FSS) [24]. The Barthel Index (BI) [25] is included to measure the level of mobility and self-care. Societal participation is assessed using the Utrecht Scale for the Evaluation of Rehabilitation–Participation (USER-P) [26]. Furthermore, the MDS-ABI contains a self-report screening question (yes/no) on experienced social support with the opportunity to elaborate/explain the given answer. The expected administration time for part B is 30 minutes [8].

### Outcome measures

#### Descriptive characteristics

Descriptive characteristics of clinicians (sex, healthcare sector, and profession) were registered upon enrolment. Demographic and injury-related characteristics of patients (age, sex, type of injury, time since injury, living situation, type of household, and level of cognitive functioning) were retrieved from the MDS-ABI to describe the sample.

#### Clinician-rated feasibility

Feasibility of the MDS-ABI as judged by the clinicians was assessed using an online questionnaire that operationalized the feasibility elements of our study, partly based on the criteria that were previously formulated for the evaluation of questionnaire feasibility [16, 17]. The measure contained questions and statements on the use according to protocol, the opinion of clinicians on the MDS-ABI, and potential barriers to implementation of the MDS-ABI in clinical practice and suggested improvements. Response options were custom made multiple-choice scales or five-point Likert scales, ranging from totally disagree (1) to totally agree (5). Furthermore, clinicians were asked to grade the overall usability of the MDS-ABI from 1–10 (1 = totally unusable in its current form, 10 = perfectly usable in its current form).

At the end of clinician-administrated part A of the MDS-ABI five questions (paper questionnaire) for the clinician were asked about filling in part A for the particular patient (the time needed to fill in part A according to file information, the administration time of the screening test, two questions on the difficulty of the questions asked, and the opportunity to add general remarks). An overview of clinician-rated feasibility measures is displayed in Table 1.

**Table 1:** Overview of outcome measures per feasibility aspect.

	Evaluation questions MDS-ABI: Part A (clinician)	Evaluation questions MDS-ABI: Part B (patient)	Feasibility questionnaire clinician	Think-aloud patient
**1. Performance of the MDS-ABI according to protocol**				
*(fidelity and dose delivered)*	x	x		
- Administration time			x	
- Instructions				
**2. Opinion on the MDS-ABI**				
*(dose received and satisfaction)*		x	x	x
- Content	x	x	x	x
- Usability			x	
- Support for the MDS-ABI				
**3. Barriers and suggested improvements** *(context)*				
- Barriers			x	
- Suggested improvements			x	

#### Patient-rated feasibility

At the end of the patient-reported questions on part B of the MDS-ABI, four questions (paper questionnaire) were asked about the patient’s opinion of the questions asked (time needed to complete all questions, opinion about the length of the questionnaire, opinion about the content of the questionnaire, and comprehensibility of the questionnaire). The patient-reported feasibility questions were inspired by the items of the QQ-10 [17], a measure that was designed to operationalize feasibility of single self-reported questionnaire use during healthcare.

For a subgroup of patients, think-aloud administration of part B of the MDS-ABI was performed. In think-aloud testing, participants are asked to verbalize their thoughts whilst using a tool or, in the case of the current study, filling in a questionnaire [27]. The method has been employed by several studies into the usability of tools [28–31] and with persons with ABI [32].

Data collection was performed by the executive researcher (AD), who is a PhD Candidate in the field of neuropsychology. The executive researcher was previously trained in conversation techniques as part of her studies. There was no relationship with most of the patients who participated in a think-aloud interview, however, three of them were familiar with the executive researcher on a professional level because of their involvement in the advisory group. During think-aloud interviews, the executive researcher administered the questionnaires while taking notes and audio-taping. No additional instructions regarding the MDS-ABI were provided to the patient during the think-aloud procedure and no non-participants were present during administration. An overview of patient-reported feasibility measures is displayed in Table 1.

### Procedure

Clinicians who were eligible for inclusion were invited by e-mail and provided informed consent when they agreed to participate, after which they received the MDS-ABI and instructions on the study protocol, such as patient inclusion criteria and informed consent procedures.

During a regular patient contact, clinicians explained the study to eligible patients and provided them with the participant information letter, the informed consent form and part B of the MDS-ABI. Patients returned the informed consent form and part B of the MDS-ABI at the next regular appointment, on average within a week. During that appointment, part A was filled in by the clinician and the MoCA was administrated.

For one of each five patients (the first patient included by every clinician), the executive researcher (AD) performed the think-aloud administration of part B of the MDS-ABI with the patient. In this case, clinicians filled in part A of the MDS-ABI about the patient and provided the contact details of the patient who agreed to participate in a think-aloud interview to the executive researcher (AD), who made an appointment for administration. The think-aloud administration was scheduled as soon as possible for the patient and took place either at the institution that patients received care at, or at the patient’s home.

### Analyses

Descriptive statistics were used for clinician and patient characteristics and for quantitative variables. For Likert-scale questions, the categories ‘totally disagree’ and ‘disagree’ as well as the categories ‘totally agree’ and ‘agree’ were merged. Qualitative data derived from open-ended questions and think-aloud interviews was clustered based on clustered based on the principles of inductive content analysis [33]. All statistical analyses were performed using SPSS version 24 [34].

## Results

### Participant characteristics

#### Clinician characteristics

**Fig 1:**
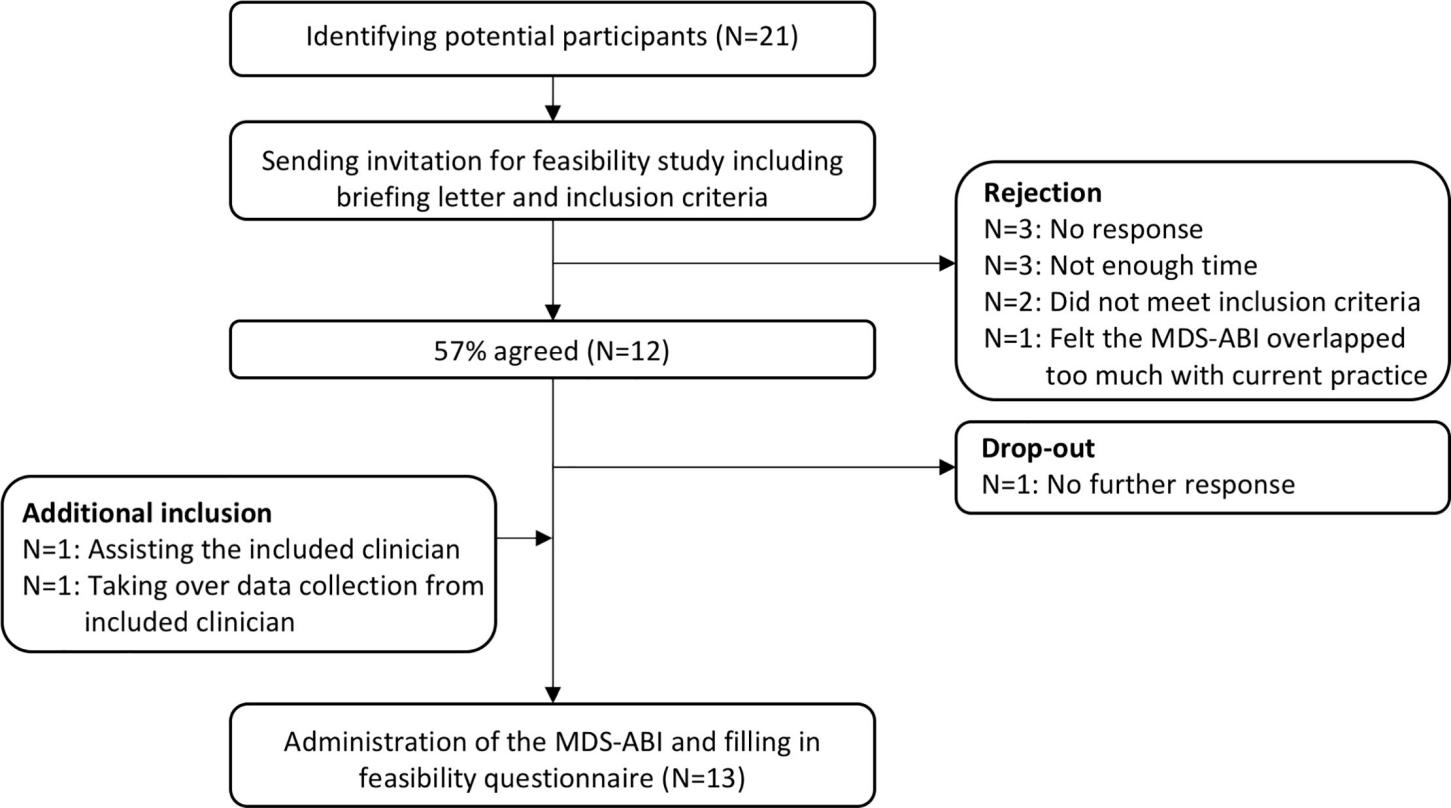
Flowchart of participating clinicians.

Twenty-one clinicians employed at institutions across healthcare sectors were approached until the desired number of clinicians (12) could be included. Twenty-one clinicians were invited. An overview of the inclusion procedure is displayed in Fig 1.

Participating clinicians were employed in a variety of healthcare sectors and disciplines (Table 2). One of the participating clinicians indicated that he/she would not see a sufficient number of patients during the study period. Therefore, a colleague offered assistance. Another clinician switched job during the study, leading to a colleague to replace him/her. The originally and additionally enrolled clinicians completed the feasibility questionnaire, leading to a total of 13 completed questionnaires.

**Table 2:** Characteristics of participating clinicians.

Characteristic	N
Sex	
*Female*	10
*Male*	3
Healthcare sector	
*Ambulatory/community-based care*	3
*Hospital*	2
*Rehabilitation care*	2
*Care for elderly persons*	2
*Mental health care*	2
*Disability care*	2
Profession	
*Psychologist/behaviourist*	6
*Allied healthcare professional/supportive staff*	6
*Physician*	1

#### Patient characteristics

**Fig 2:**
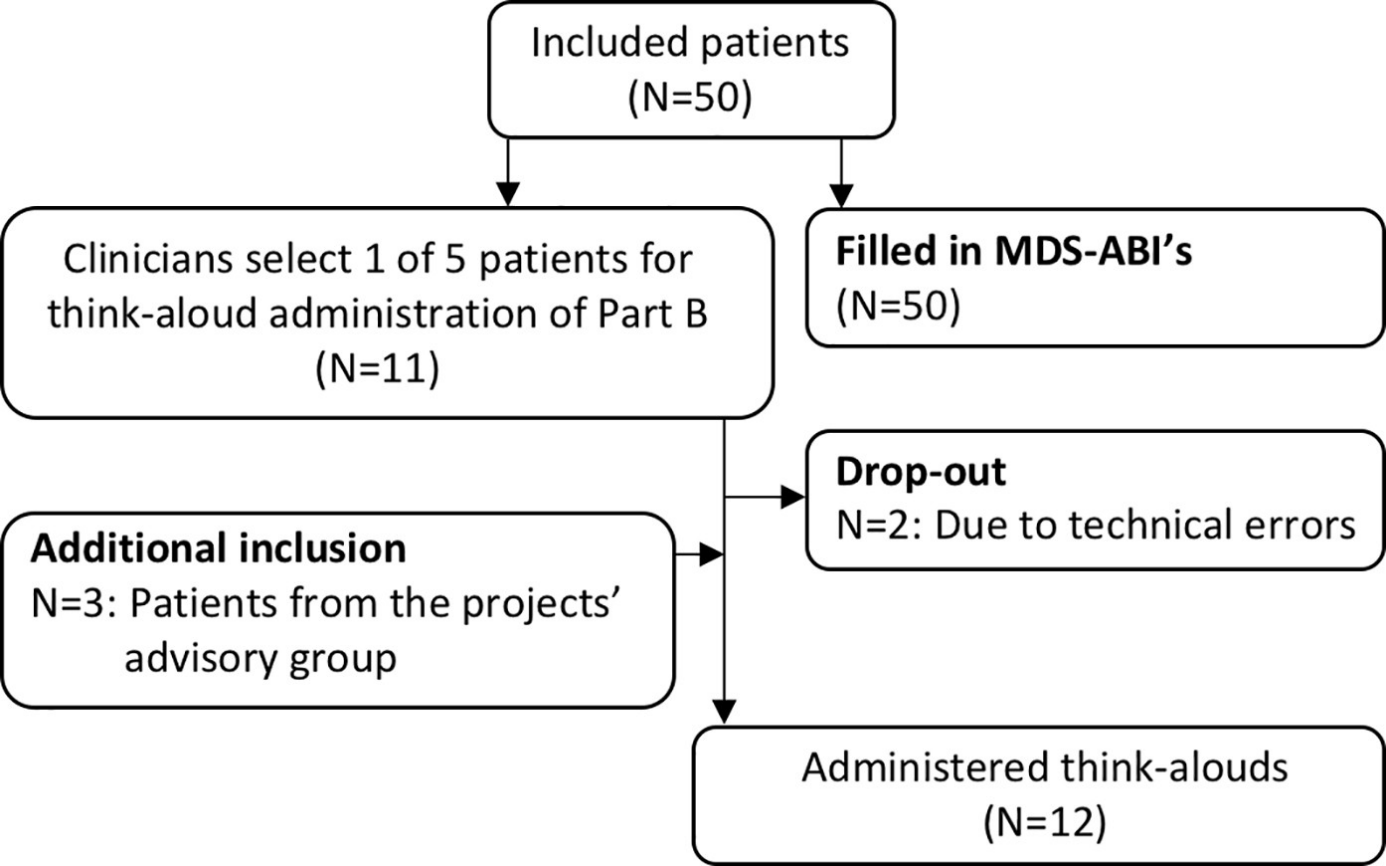
Flowchart of participating patients.

We initially asked each clinician to include five patients (55 patients in total). Due to a lack of time, some clinicians included less than five patients, leading to a total of 50 patients included in the current study. Eleven patients were selected for a think-aloud administration of part B. Two of the think-aloud administrations failed. Therefore, three members of the client panel (which has been involved in setting up and conducting the study from the start) participated in a supplementary think-aloud interview, leading to a total of 12 administered think-alouds (Fig 2).

The mean age of the patients was 55.2 years (SD = 17.5) and 54% (n = 27) was male. Most patients had suffered a stroke (n = 33, 69%), on average 6.9 years ago (SD = 9.8). The majority of patients lived independently (n = 41, 82%), i.e. not in a residential facility, and most of them lived together with others (n = 31, 62%). The mean score on MoCA was 24.5 (sd = 4.8) and 46% (n = 22) of the patients scored below the cut-off (26) for normal cognitive functioning. Patient characteristics are summarised in Table 3.

**Table 3:** Characteristics of patients included in feasibility study.

Characteristic	N	M ± SD (range) or n (%)
Mean age in years ± SD (range)	48	55.2 ± 17.5 (22.2–98.5)
Sex male, n (%)	50	29 (58)
Type of injury, n (%)	49	
*CVA*		34 (69.4)
*TBI*		7 (14.3)
*Other*		7 (14.3)
*Unknown*		1 (2.0)
Time since injury in years ± SD (range)	47	6.9 ± 9.8 (0.1–22.2)
Living situation, n (%)	50	
*Independent*		41 (82.0)
*Not independent*		9 (18.0)
Type of household, n (%)	50	
*With others*		31 (62)
*Alone*		19 (38)
Cognitive functioning (MoCA) score ± SD (range)	48	24.5 ± 4.8 (12–30)

Percentages are rounded so may not total to exactly 100%. CVA = cerebrovascular accident, TBI = traumatic brain injury, MoCA = Montreal Cognitive Assessment.

### Performance according to protocol

The reported administration time of part A of the MDS-ABI was 30.9 minutes on average (SD = 15.8), of which the administration of the cognitive screener (MoCA) took 17.7 minutes (SD = 6.8). Patients needed 23.9 minutes on average (SD = 12.0) for filling in part B.

Of all 13 clinicians, 10 reported to always have administered all elements of part A of the MDS-ABI. Clinicians indicated that they sometimes did not have sufficient information to complete the Barthel Index and that the CIRS was incomprehensible. Eight out of 13 clinicians reported to have used the MDS-ABI as prescribed in the instructions. Explanations for deviating from the instructions were forgetting to ask about years of education, and abbreviating the instructions that were believed to be too comprehensive. Further, some clinicians experienced problems with printing the MDS-ABI, leading to a distorted lay-out.

### Opinions of patients and clinicians

#### Content

The majority of both clinicians (11 out of 13) and patients (n = 38, 79%) considered their part of the questionnaire to be of adequate *length*. The majority of the clinicians considered the *instructions* of part A and part B clear (11 out of 13) (Table 4). During think-aloud administration, patients mentioned they usually skip large pieces of instruction texts. As reading can be strenuous, they indicated they would have benefited from a visual representation of the general instructions. Concerning *composition* of the MDS-ABI, most clinicians reported that the lay-out of the MDS-ABI was clear (8 out of 13), that it contained relevant domains (11 out of 13), and suitable measurement instruments (8 out of 13) (Table 4). When patients were asked if they thought the MDS-ABI contained the right questions to obtain an accurate overview of their current status, the majority (n = 25, 54%) agreed. Reasons for patients to answer ‘partly’ (n = 20, 44%) or ‘no’ (n = 1, 2%) were a reported lack of context that was needed to complete some questionnaires, such as a definition of the response categories that reflected frequency, i.e. ‘often’, the complex 7-point Likert scale of the FSS, and the fact that the questionnaires focus on experienced restrictions. Patients who participated in think-aloud administration remarked they appreciated the possibility to answer an open-ended question at the end of the questionnaire, in case there was an important topic that was not covered by the MDS-ABI.

**Table 4:** Clinician (N = 13) answers to statements regarding the content and usability of the MDS-ABI.

Statement (CR)	Disagree	Neutral	Agree
	(n)^a^	(n)	(n)^b^
The goal of the MDS-ABI is clear to me	0	0	13
The instructions of part A are clear to me	1	1	11
The instructions of part B are clear to persons with ABI	0	2	11
The lay-out of the MDS-ABI is clear	2	3	8
The MDS-ABI contains relevant domains	1	1	11
The MDS-ABI contains suitable measurement instruments	0	5	8
The MDS-ABI is appropriate for use in healthcare settings	1	4	8
The MDS-ABI is appropriate for use in research settings	0	5	8

^a^ Categories “totally disagree” and “disagree” were collapsed.

^b^ Categories “totally agree” and “agree” were collapsed.

#### Usability

The majority of the clinicians rated the MDS-ABI in its current form *appropriate for use in healthcare settings as well as for research purpose*s (8 out of 13, both) (Table 4).

The majority of patients (n = 35, 80%) reported the questions in part B were *comprehensible*. During think-aloud administrations, patients indicated the question about living situation should be rephrased. Further, think-aloud administration of the HADS indicated it was unclear to patients whether the questions which referred to the past (for example, “not as much as I used to do”), were aimed at “before I acquired the brain injury” or “before the past week”. In addition, with the FSS, patients said to have missed questions about mental fatigue. Regarding the USER-P, patients indicated they found it difficult to calculate the activities they had performed in the past four weeks. Moreover, they remarked that their answer to the items of the restrictions and satisfaction subscales would differ depending on the example they answered it for.

With the evaluation question at the end of part A of the MDS-ABI, clinicians reported that *part A was easy to administer for the particular patient* (n = 25, 52%), or partly easy (n = 23, 48%). Main reasons that were given were that the CIRS was difficult and time consuming to administer, supported by secondary analysis of missing values (n = 8, 16%). Those clinicians who explained why they found the CIRS difficult to understand, said they found it difficult to find all required information in the patient file, especially when they themselves did not have a medical background. Further, medical files often appeared to be incomplete. When clinicians were asked if they thought part B was easily filled in by patients, they answered ‘yes’ for the majority of patients (n = 34, 74%). For some patients, clinicians estimated that part B was difficult to complete due to patients’ cognitive problems, aphasia or illiteracy, fatigue, perceptual problems, impaired awareness, or being in the subacute phase of the injury or having been hospitalised for a longer period which complicates the administration of items that ask about everyday life. Overall, clinicians rated the usability of the current form of the MDS-ABI a 7.0 out of 10 (SD = 0.6).

#### Clinician and patient support for the MDS-ABI

All clinicians reported to understand the aim of a minimal dataset (n = 13). When judging the added value of the MDS-ABI to the sector they are employed in, clinicians estimated that it would be of some (n = 10) or much (n = 3) added value and that they would definitely (n = 4) or potentially (n = 8) employ use the MDS-ABI in their future clinical work. Clinicians would use the MDS-ABI to facilitate the intake process and treatment plans, with monitoring and accordingly adjusting treatments, for diagnostic and for research purposes. Clinicians who indicated they might or would not use the MDS-ABI in the future, explained they thought the MDS-ABI in its current form was not suitable for the entire population, administration was too time-consuming, the selected measurement instruments were not suitable, other measurement instruments were standard practice at the institution, or some parts of the MDS-ABI were regarded specific to one healthcare discipline.

During think-aloud, patients indicated their answers are heavily dependent on the phase they are in, and that it would be insightful to monitor their changes over time. Moreover, they remarked that these were questions he/she had repeatedly filled in before and they mentioned that the questions were confrontational, but also offered insight into their own functioning.

### Barriers and suggested improvements

The majority of clinicians (7 out of 13) experienced some barriers during the administration of the MDS-ABI: patients being incapable of writing, patients having trouble completing the self-report questionnaires because of aphasia, fatigued patients, patients being emotionally overwhelmed because of confronting questions and clinicians lacking experience with the MoCA, resulting in the need to refer to the manual multiple times.

Regarding improvements, clinicians suggested a more attractive lay-out, a digital version featuring an option to let the device read questions out loud, clearer instructions, and adjusting the wording of the questionnaires to suit the capabilities of persons with intellectual disabilities, aphasia, or cognitive problems. Further, clinicians suggested to add a measure of caregiver strain such as the Caregiver Strain Index (CSI) [35] to the MDS-ABI, to replace the CIRS with a less complicated approach to detailing medical information, to replace the MoCA with a cognitive screener that is more sensitive to subtle cognitive impairment, and to leave out the USER-P, because it was not suitable for use in a post-acute setting.

## Discussion

In the current study, the MDS-ABI was assessed for feasibility among 13 clinicians and 50 patients within six healthcare sectors. In general, the MDS-ABI performed according to protocol to a large extent. Both patients and clinicians were overall satisfied with the content of the MDS-ABI. Regarding the usability, some patients indicated there was a lack of context for some questionnaires and some suggested to alter the wording or items of a number of the questionnaires. Furthermore, clinicians experienced problems with the CIRS, which they considered difficult and time-consuming to administer. In addition, clinicians indicated that the MDS-ABI would not be suitable for all ABI-patients because of potential cognitive problems, communicative problems, fatigue, perceptual problems, or impaired awareness of deficits. The MDS-ABI was considered appropriate for use in research as well as healthcare settings by clinicians. Overall, clinicians indicated they would potentially use the MDS-ABI in future. However, the rating of 7.0 of the overall usability of the MDS-ABI in its current form, the high number of clinicians who found it only partly easy to administer, and the fact that most clinicians think the MDS-ABI has only some added value to clinical practice, indicates the need to adjust the MDS-ABI before dissemination.

To improve the usability of the MDS-ABI, the CIRS was replaced with a screening question [36]. The suggestion to drop the MoCA and replace it with a screener that is more sensitive to subtle cognitive impairment could not be implemented as, to our knowledge, no such instrument is currently available for ABI-patients [37]. At the moment, we believe it is inappropriate to modify the content of the validated questionnaires, or adding questions or response options to them, as it would alter their validity [38]. Also, all of the above questionnaires were validated with ABI-patients in previous studies [39–41] and none of the issues described by the patients had an effect on the validity of the scales. Nonetheless, in 2011, Lerdal and Kottorp [42] reported a better performance of the FSS with stroke patients when the first two items of the scale were removed. Because the MDS-ABI is aimed at patients with all types of brain injury, the MDS-ABI for now will feature the original, 9-item FSS until the validity of the 7-item FSS is assessed in other populations, such as patients with traumatic brain injury. Meanwhile, the 7-item score can be calculated using the MDS-ABI data, may potential users wish to do so.

Those clinicians who would not (certainly) use the MDS-ABI in the future, mainly indicated it was not suitable for the entire patient group and that administering part A was too time-consuming. With regard to administration duration, the CIRS has the longest administration time of the questions in part A. We expect its replacement with a screening question will significantly reduce the administration time of part A. Last, as the USER-P asks about societal participation in the foregoing weeks, provided that these were “average” weeks, it proved unsuitable for use with patients in the acute phase of the injury. Therefore, instructions were added to omit this part of the questionnaire if the patient is admitted to the hospital at the time of administration.

The MDS-ABI was developed for use with every person with ABI, but appeared unsuitable for a proportion of the target group due to communicative or cognitive limitations. Oftentimes, patients who experience these limitations are excluded from assessments in the context of scientific research or health care, when in fact, these patients are most prone to more severe long-term consequences of brain injury [43–48], leading to bias in research [49]. In order to be able to capture core data on the full spectrum of persons with ABI, we now developed a proxy-rated version of the self-reported part of the MDS-ABI (part B), which can be completed by someone who is close to the patient. As it can be complicated to determine a patient’s capability to self-report prior to administering the MDS-ABI, we advise for this proxy-module to be administered with all patients. Furthermore, clinicians expressed the need for information about the informal caregiver of patients with ABI. In response, we developed an additional module for the MDS-ABI that obtains information of the caregiver status.

In order to enhance user experience, improvements will be made regarding the lay-out of the pen-and-paper MDS-ABI. Suggested rewording of questions will be implemented and general instructions will be visualised as far as possible, as pictures that are linked to written text can increase attention and recall of information [50, 51]. Possibilities to develop a digital version of the MDS-ABI are currently being explored. Remarks of patients regarding their wish to monitor their own change over time indicates they could benefit from a summary of the results to take with them. Opportunities for developing such a summary will be explored.

With replacing the CIRS and the addition of a proxy-module, making the MDS-ABI more suitable for all ABI-patients, we expect to have resolved most issues, to have decreased the number of potential barriers of using the MDS-ABI, and to stimulate its future implementation. Public involvement, such as governmental recommendations [52], or support of large professional and patients’ organisations in the field of ABI, may enhance future use of the MDS-ABI.

### Strengths and limitations

One of the strengths of our study is the diverse range of methods used. Furthermore, the inclusion of patients besides clinicians has shed light on different perspectives on the MDS-ABI, as is increasingly recommended by research authorities [53]. The inclusion of patients and clinicians from relevant healthcare sectors and clinicians from a variety of healthcare disciplines gave an estimation of the performance of the MS-ABI in a wide range of settings. The fact that patients remarked that they repeatedly had answered questions like the MDS-ABI before underlines the need for standardization of data collection to relieve the need for patients to ‘repeat their story’ when moving through the healthcare chain. Further, answering the MDS-ABI provided insight into the patients’ own functioning, indicating its use can be beneficial to patients as well as healthcare professionals, as was previously found in research into the feasibility of patient reported outcome sets [54].

The current study was limited by clinicians lacking time to include the intended number of patients, which, in itself, can be indicative of the feasibility of the MDS-ABI. Moreover, the fact that this study employed ‘being capable of filling in a self-report questionnaire’ as one of the inclusion criteria resulted in a pre-selection of patients, which could have overestimated the actual usability of the MDS-ABI for the entire patient group. Notwithstanding, clinicians raised their concern about applicability of the MDS-ABI to patients who are incapable of self-report.

One could debate about the ideal sample size for feasibility studies in general, though we have attempted to include as many patients and clinicians to obtain good insight in the feasibility of the MDS-AB, without posing a burden on too many patients and healthcare staff. Nevertheless, our results should be interpreted in light of the limited sample size. As we only included one physician, it is possible that we have missed valuable additional insights from other physicians. However, in the Netherlands, physicians frequently coordinate rehabilitation teams, whereas other types of healthcare professionals are responsible for everyday care for people with brain injury (especially outside the hospital). Therefore, in everyday healthcare, these types of measurement instruments are predominantly administered by psychologists and/or allied healthcare professionals such as occupational therapists. To reach the intended user group, we focused on these groups of clinicians when inviting participants. Moreover, for the other included professions accounts that the opinions of the different professionals within one profession showed great similarities. The majority of our clinician sample was female, which can be explained by the fact that about three thirds of the allied healthcare professionals and psychologists in the Netherlands is female [55].

One threat to the validity of feasibility studies is a potential social desirability bias [56]. Because of practical reasons (being able to trace whether clinicians had filled in the online questionnaire and the MDS-ABI containing demographical information), none of the feasibility measures were anonymous. However, involved clinicians had no personal connection to the executive researcher (AF) and during think-aloud administration, patients were encouraged to speak their mind as their honest opinion would only improve the MDS-ABI.

## Recommendations

The results of this feasibility study can be applied to other initiatives aimed at developing minimal datasets. For instance, this study showed that the development process of minimal datasets should involve clinicians from the very beginning in order to consider the clinical feasibility throughout the process. Further, this study indicated that minimal datasets should correspond with measures that are already used in the field, and feature a digital version, consistent with the previous finding that a digital application leads to a greater ease of administration of minimal datasets [57]. Previous research emphasized the importance of incorporating patient opinions into health evaluations [58]. However, patients can be incapable of self-report for a variety of reasons, such as communication problems or disorders of consciousness. In order to stimulate involvement of patients in clinical decision making, we recommend including PROMs or, in the case of incapability to self-report, proxy-reported measures in minimal datasets to obtain an indication of the patients’ perspectives. The use of a minimal dataset in general can streamline data collection to enhance data comparability and stimulate to combine datasets for healthcare as well as research purposes [59]. This study showed that clinical applications of a minimal dataset can include assisting the intake process, aiding treatment planning, and monitoring patients. Further, patients indicated that (periodically) completing the MDS-ABI can help to gain insight into their own recovery process.

## Conclusion

In conclusion, the MDS-ABI is a promising tool for obtaining core information on brain injury patients across disciplines and healthcare settings. Although the present study concerns a Dutch minimal dataset, all instruments are valid for use in brain injury samples and are widely used internationally. Further, the MDS-ABI will be made available in English to enhance international usability. The MDS-ABI was adjusted according to the recommendations made by patients and clinicians, after which we expect the MDS-ABI to be feasible for use in a large proportion of healthcare settings and for most ABI-patients. For patients who are incapable of self-report, a proxy-reported version of the self-reported part B was developed. Additionally, a module for measuring the status of the informal caregiver was developed. Future directions should be aimed at involving professional and patients’ organisations in the implementation of the MDS-ABI, enhancing its future use.

## Supporting information

S1 Appendix Outcome measures. (DOCX)

S2 Appendix Supplementary tables results on clinician-rated (CR) and patient-rated feasibility aspects. (DOCX)
