# Supplementary material for: Feasibility of a minimal dataset for adults with acquired brain injury in Dutch healthcare practice
Source: PLoS One. 2020 Jun 22;15(6):e0235085. doi: 10.1371/journal.pone.0235085 (PMC7307757; doi:10.1371/journal.pone.0235085)
Supplement: S1 Appendix — (DOCX) [file pone.0235085.s001.docx]

**S1 Appendix. Outcome measures.**

**1. Feasibility questionnaire (clinicians)**

| Name: |  |
| --- | --- |
| Function: |  |
| Organisation: |  |

Healthcare professional

1. This questionnaire consists of a number of statements. Can you indicate to what extent you agree or disagree with these statements?

|  | **Totally disagree** | **Disagree** | **Neutral** | **Agree** | **Totally agree** |
| --- | --- | --- | --- | --- | --- |
| 1. The goal of the MDS-ABI is clear to me | 1 | 2 | 3 | 4 | 5 |
| 1. The instructions of part A are clear to me | 1 | 2 | 3 | 4 | 5 |
| 1. The instructions of part B are clear to persons with ABI | 1 | 2 | 3 | 4 | 5 |
| 1. The lay-out of the MDS-ABI is clear | 1 | 2 | 3 | 4 | 5 |
| 1. De MDS-ABI contains relevant domains | 1 | 2 | 3 | 4 | 5 |
| 1. De MDS-ABI consists of suitable measurement instruments | 1 | 2 | 3 | 4 | 5 |
| 1. De MDS-ABI is appropriate for use in healthcare settings | 1 | 2 | 3 | 4 | 5 |
| 1. De MDS-ABI is appropriate for use in research situations | 1 | 2 | 3 | 4 | 5 |
| **Explanation** (optional)**:** | | | | | |
| **6.**  **7.** | | | | | |

1. What is your opinion on the length of the MDS-ABI?

- Too long
- Too short
- Just right

1. Have you administered all parts of the MDS-ABI? *If not, please indicate which parts you did not administer and for what reason.*

- Yes
- No, because:

1. Have you used the MDS-ABI as prescribed in the instructions? *If you have (partially) deviated from the instruction, please indicate which questions you deviated from and for what reason.*

- Yes
- Partially
- No

Explanation:

1. Was part A (healthcare professional) of the MDS-ABI easy to complete for you? *If partially or if not, could you indicate which parts were difficult to complete and why?*

- Yes
- Partially
- No

Explanation:

1. Was part B (person with ABI) of the MDS-ABI easy to complete for persons with ABI? *If partially or if not, could you indicate which parts were difficult to complete and why?*

- Yes
- Partially
- No

Explanation:

1. Are there (other) factors that have complicated the administration of the MDS-ABI? *If yes, could you indicate as specifically as possible which factors?*

- No
- Yes, namely:

1. Would you use the MDS-ABI in your future clinical practice?

- Yes
- Maybe
- No

1. For which purposes would you use the MDS-ABI? (multiple choices possible)

- At intake (to organise the care process)
- Diagnostics
- To follow and adjust treatments
- For scientific research
- Not applicable, I would not use the MDS-ABI
- Other, namely:

1. Why would you (potentially) not use the MDS-ABI? (multiple choices possible)

- Not applicable: I am going to use the MDS-ABI
- Administration takes too much time
- I consider the selected measurement instruments unsuitable
- Other measurement instruments are standard practice at the institution work
- I think that (a proportion of the) persons with ABI are unable to complete the MDS-ABI
- Other, namely:

1. According to you, how could the MDS-ABI be improved? (multiple choices possible)

- Not, I would not change the MDS-ABI
- A clearer instruction for healthcare professionals
- A clearer instruction for people with ABI
- A more attractive lay-out
- Make the MDS-ABI available in a digital format
- Deleting the following measurement instruments/variables:

| **Part A: healthcare professional** | **Part B: person with ABI** |
| --- | --- |
| - General information - HADS - Screening question social support - FSS - SF-12 - USER-P | - Injury characteristics - Screening questions communication - MoCA - CIRS - Barthel Index |

- Adding the following tools/variables:
- Other, namely:

1. What score would you give the usability of the MDS-ABI?

___ (0-10)

1. To what extent do you think the MDS-ABI has added value within the sector in which you work?

- Much added value
- Some added value
- No added value

Explanation:

1. Do you have any (other) recommendations for improving the MDS-ABI?

**2. Evaluation questions part A (clinician)**

1. How long did it take you to complete your part of the questionnaire (Part A), excluding the administration of the MoCA, for this particular person?

__ minutes

1. How long did the administration of the MoCA take for this particular person?

__ minutes

1. Was this part (Part A, healthcare professional) easy to administer for this particular person?

- Yes
- Partially
- No

1. Do you think that part B (person with brain injury) was easy to administer for this person?

- Yes
- Partially, because:
- No, because:

1. Do you have any other comments/additions?

**3. Evaluation questions part B (patient)**

1. We have asked you a number of questions. Would you like to tell us something else you think is important about yourself?
2. How long did it take you to complete this questionnaire?

__ minutes

1. What did you think of the length of this questionnaire?

- Too long
- Too short
- Just right

1. Did you think the right questions were asked to get a good picture of you?

- Yes
- Partially
- No

**Explanation:**

1. Has anyone assisted you in completing this questionnaire?

- No, I completed the questionnaire alone.
- Yes, someone else wrote down the answers; I chose the answers myself.
- Yes, I chose and noted the answers with someone else.
- Someone else chose and wrote down the answers for me.

1. Was this questionnaire easy to understand for you?

- Yes
- Partially
- No
