# Supplementary material for: Feasibility of a minimal dataset for adults with acquired brain injury in Dutch healthcare practice
Source: PLoS One. 2020 Jun 22;15(6):e0235085. doi: 10.1371/journal.pone.0235085 (PMC7307757; doi:10.1371/journal.pone.0235085)
Supplement: S2 Appendix — (DOCX) [file pone.0235085.s002.docx]

**S2 Appendix. Supplementary tables results on clinician-rated (CR) and patient-rated feasibility aspects.**

**Table 1**. Clinician (CR) and patient (PR) estimates of administration times of the MDS-ABI

| **Variabele** | **N** | **M** | **SD** |
| --- | --- | --- | --- |
| Mean administration time part A without MoCA (CR) | 48 | 15.2 | 9.0 |
| Mean administration time MoCA (CR) | 46 | 17.7 | 6.8 |
| Mean administration time part B (PR) | 44 | 23.9 | 12.0 |

**Table 2.** Clinician (CR) and patient (PR) answers to questions regarding length of the MDS-ABI

| **Variable** | **N** | **Too long (%)** | **Exactly right (%)** | **Too short (%)** |
| --- | --- | --- | --- | --- |
| What do you think of the total length of the MDS-ABI? (CR) | 13 | 8% | 85% | 8% |
| What do you think of the length of part B of the MDS-ABI? (PR) | 42 | 19% | 79% | % |

**Table 3.** Clinician answers to statements regarding the content and usability of the MDS-ABI

| **Statement (CR)** | **N** | **Totally disagree**  **(%)** | **Dis-agree**  **(%)** | **Neutral**  **(%)** | **Agree**  **(%)** | **Totally agree**  **(%)** | **Med** | **IQR** |
| --- | --- | --- | --- | --- | --- | --- | --- | --- |
| The goal of the MDS-ABI is clear to me | 13 | 0 | 0 | 0 | 46 | 54 | 5 | 1 |
| The instructions of part A are clear to me | 13 | 0 | 8 | 8 | 77 | 8 | 4 | 0 |
| The instructions of part B are clear to persons with ABI | 13 | 0 | 0 | 15 | 77 | 8 | 4 | 0 |
| The lay-out of the MDS-ABI is clear | 13 | 0 | 15 | 23 | 62 | 0 | 4 | 1 |
| The MDS-ABI contains relevant domains | 13 | 0 | 8 | 8 | 85 | 0 | 4 | 0 |
| The MDS-ABI contains suitable measurement instruments | 13 | 0 | 0 | 38 | 54 | 8 | 4 | 1 |
| The MDS-ABI is appropriate for use in healthcare settings | 13 | 0 | 8 | 31 | 62 | 0 | 4 | 1 |
| The MDS-ABI is appropriate for use in research settings | 13 | 0 | 0 | 38 | 54 | 8 | 4 | 1 |

**Table 4.** Clinician answers to fidelity questions

| **Question** | **N** | **Yes (%)** | **Partly (%)** | **No (%)** |
| --- | --- | --- | --- | --- |
| Have you administered all elements of part A? (CR) | 13 | 77 | N/A | 23 |
| Have you used the MDS-ABI according to the instructions? (CR) | 13 | 62 | 38 | 0 |

**Table 5.** Patient rated questions regarding usability

| **Question** | **N** | **Yes (%)** | **Partly (%)** | **No (%)** |
| --- | --- | --- | --- | --- |
| Does the MDS-ABI contain the right questions to obtain an accurate overview of your current status? (PR) | 46 | 54 | 43 | 2 |
| Were the questions easy to understand for you? (PR) | 44 | 80 | 20 | 0 |

**Table 6.** Clinician rated questions regarding usability of the MDS-ABI, on patient level and in general

| **Question** | **N** | | **Yes (%)** | | **Partly (%)** | **No (%)** |
| --- | --- | --- | --- | --- | --- | --- |
| Was part A of the MDS-ABI easy to fill in for this particular patient? (CR) | 48 | | 52 | | 48 | 0 |
| Was part A of the MDS-ABI, in general, easy to administer? (CR) | 13 | | 38 | | 54 | 8 |
| Do you think that part B is easily filled in for this particular patient? (CR) | | 46 | | 74 | 22 | 4 |
| Do you think part B is generally easily filled in by ABI-patients? (CR) | | 13 | | 38 | 54 | 8 |

**Table 7.** Clinician answers to support question

| **Question** | **N** | **High added value (%)** | **Some added value (%)** | **No added value (%)** |
| --- | --- | --- | --- | --- |
| Do you think the MDS-ABI is of added value to your healthcare sector? (CR) | 13 | 23 | 77 | 0 |

**Table 8.** Clinician answers to question on future use

| **Question** | **N** | **Yes (%)** | **Maybe (%)** | **No (%)** |
| --- | --- | --- | --- | --- |
| Would you use the MDS-ABI in your future practice? (CR) | 13 | 31 | 62 | 8 |

**Table 9.** Clinician rated barriers to the administration of the MDS-ABI

| **Question (CR)** | **N (%)** | **Yes (%)** | **No (%)** |
| --- | --- | --- | --- |
| Are there any barriers to the administration of the MDS-ABI? | 13 | 54 | 46 |
